# ‘THEY SAY ISLAM HAS A SOLUTION FOR EVERYTHING, SO WHY ARE THERE NO GUIDELINES FOR THIS?’ ETHICAL DILEMMAS ASSOCIATED WITH THE BIRTHS AND DEATHS OF INFANTS WITH FATAL ABNORMALITIES FROM A SMALL SAMPLE OF PAKISTANI MUSLIM COUPLES IN BRITAIN

**DOI:** 10.1111/j.1467-8519.2011.01883.x

**Published:** 2011-06-07

**Authors:** ALISON SHAW

**Affiliations:** University of Oxford

**Keywords:** Islam, abortion, withdrawal of life support, stillbirth, infant death, British Pakistanis

## Abstract

This paper presents ethical dilemmas concerning the termination of pregnancy, the management of childbirth, and the withdrawal of life-support from infants in special care, for a small sample of British Pakistani Muslim parents of babies diagnosed with fatal abnormalities. Case studies illustrating these dilemmas are taken from a qualitative study of 66 families of Pakistani origin referred to a genetics clinic in Southern England. The paper shows how parents negotiated between the authoritative knowledge of their doctors, religious experts, and senior family members in response to the ethical dilemmas they faced. There was little knowledge or open discussion of the view that Islam permits the termination of pregnancy for serious or fatal abnormality within 120 days and there was considerable disquiet over the idea of ending a pregnancy. For some parents, whether their newborn baby would draw breath was a main worry, with implications for the baby's Muslim identity and for the recognition of loss the parents would receive from family and community. This concern sometimes conflicted with doctors' concerns to minimize risk to future pregnancies by not performing a Caesarean delivery if a baby is sure to die. The paper also identifies parents' concerns and feelings of wrong-doing regarding the withdrawal of artificial life-support from infants with multiple abnormalities. The conclusion considers some of the implications of these observations for the counselling and support of Muslim parents following the pre- or neo-natal diagnosis of fatal abnormalities in their children.

## INTRODUCTION

How do parents respond when doctors diagnose a fatal abnormality in a pregnancy or newborn baby? What ethical dilemmas arise for them, and how do they negotiate the choices they face? This paper presents three issues of ethical concern for British Pakistani Muslim parents following the medical diagnosis in pregnancy, or at or soon after birth, of abnormalities considered incompatible with life. These issues concern the termination of pregnancy, the management of childbirth and the withdrawal of life support from infants in special care. After providing some details of the qualitative study within which these issues emerged as concerns for parents, this paper presents three case studies, each illustrating one of these ethical issues, which can be summarized as three questions:

- Is it permissible to end the pregnancy?
- If we continue with the pregnancy, should we try to ensure that the baby will breathe?
- After the birth, can we withdraw life support from a baby in intensive care?

Each case presentation is followed by a discussion that attempts to situate each dilemma in its local context and asks to what extent the dilemma reflects a general question within Islamic bioethics and to what extent it reflects Islam as understood by the study participants, in relation to their particular social and cultural background. The concluding section reflects on the significance of these dilemmas for parents and on the implications for medical service provision.

Within the UK, supporting informed decision-making is recognized as an important principle of good patient care. This paper identifies gaps where improvements could be made in ensuring patients are provided with appropriate information, support and practical advice in the decisions they make. It also identifies areas where additional information and advice would be of value to doctors and medical staff responsible for patient care. It is important to emphasize that the dilemmas described here are unlikely to be experienced in the same way by all Muslims, because what is ‘customary’ and what is ‘religious’ varies with ethnic and social background, school of Islamic thought, and between individuals. The issues identified here reflect the concerns of a small sample of British parents with family origins in Mirpur district in Azad Kashmir. On the other hand, the majority of Britain's 1.6 million Muslims are of South Asian heritage, many of them of Pakistani origin and specifically from Azad Kashmir, and so the issues raised here are likely to have general relevance for medical service provision in the UK as well as in other multi-ethnic societies where Muslims are a minority.

## BACKGROUND

The case studies are drawn from a qualitative study of the experiences of British Pakistani Muslim couples who have had a pregnancy, baby or child affected with a genetic condition.^1^ A total of 66 couples participated in this study. Most participants were recruited from the Pakistani referrals to the genetics clinic of a hospital in a town in England.^2^ The town is located in a district that contains the second largest Pakistani population in southern England outside London. The majority of the district's Muslim population (of 10,527 or 6.5% of the total district population) comprises Pakistanis, who number 9,703 or 6% of the district population and share the broad demographic and socio-economic features of British Pakistanis generally. As in other British cities where Pakistanis have settled in significant numbers, so too in the study population most Pakistanis originate from Mirpur district in Azad Kashmir.

In most couples in this study, one partner was British-born and educated at least to General Certificate of Education level (GCSE) or equivalent, while their spouse was raised in Pakistan and came to Britain for or following marriage. Most couples (61/66) were also consanguineously related, as first cousins or more distantly. Their marriages had been arranged by their families, as is conventional in Pakistan. The practice of arranged transnational marriages with consanguineous kin has been an important feature of the socio-economic links that British Pakistani families maintain with relatives in Pakistan.

A dominant discourse of disapproval has arisen in Britain regarding the practice of arranged marriages with cousins from Pakistan.^3^ This is based on three perceptions. One is the common but problematic assumption that arranged marriage is equivalent to forced marriage, raising human rights concerns. The second is that the practice encourages insularity and inhibits integration.^4^ The third is associated with evidence of an elevated risk of recessive genetic conditions (those caused by inheriting two copies of a gene carrying a mutation) in children born to consanguineous parents. Among the study participants, there was widespread scepticism of the discourse of elevated recessive risk in consanguineous marriages, this being perceived as discriminatory in singling out a particular population on the basis of its marriage pattern.^5^

The problems observed in babies and children of the adults in this study reflect the background risk of genetic conditions shared with the general population plus the elevated risk of recessive conditions associated with parental consanguinity. Overall, 69 different conditions were observed in 66 families; three couples had children who were affected by two different conditions. The majority of the conditions were autosomal recessives (44/69) of widely varying types, and some of these were fatal, resulting in miscarriage, stillbirth or in neonatal death.

On the basis of observation of genetics' consultations and interviews conducted by the author in participants' homes, the research sought to explore parents' understandings of illness causality and inheritance, attitudes towards the management of risk in subsequent pregnancies, and risk communication within the wider family.^6^ During interviews and observations, the experience of infant death at birth or soon afterwards emerged as a central concern for some parents. Ten couples had between them lost eighteen babies, who were either stillborn or who died at or soon after birth. All but one or two deaths were caused by lethal recessive conditions. Five couples experienced infant deaths two or three times from the same condition. One couple had three infants die from two different recessive conditions. All but one of these infant deaths had been predicted by doctors, who diagnosed the fatal condition prenatally (in 12/18 cases) or in a newborn baby (in 5/18 cases). In just one case, the infant's death took place unexpectedly at nine months and the diagnosis of a recessive genetic condition was made afterwards.

For these parents, the medical foretelling of infant death raised dilemmas concerning the correct thing to do as Muslims. In pregnancy, it raised the question of the permissibility of abortion in Islam. For parents who continued with the pregnancy, it raised a concern about whether or not the baby would breathe and therefore be identified as Muslim. For the parents of babies who survived birth but were kept alive by technological means, it raised questions concerning the permissibility of withdrawing life support from a baby in intensive care. Each of these dilemmas and parents' responses to it is illustrated below with a case study and a discussion of how far the issue represents a problem of ‘Islamic bioethics’ or a social or cultural matter that reflects Islam as it is understood by the participants in this study.

## CASE 1

This case concerns the first pregnancy of a UK-raised woman called Shakeela (not her real name) and her husband who is her cousin from Pakistan. At the 20-week ultrasound scan, doctors noted that the foetus showed ‘water on lungs’, explained as indicative of a serious abnormality, and raised the question of terminating the pregnancy. The couple continued with the pregnancy, and the 24 week scan showed more water on the lungs and an abnormal heart. By this time, the 24 week limit for medical abortion had been reached. The next two scans continued to show fluid on the brain. Since it was now too late for a medical abortion, the doctors raised the option of ending the pregnancy by feticide (an injection to kill the foetus) and an induced labour.

The couple proceeded with the pregnancy and the doctors explained that the abnormalities indicated that a normal birth would be impossible. The baby had an adult-sized head as a result of the liquid on the brain. The doctors considered performing a Caesarean section (removing the baby after surgical incision into the abdomen and uterus) but explained that this would require two incisions into the abdomen – one across and one along – rather than the usual single incision, increasing the risk of complications for mother and baby, and for the mother in subsequent pregnancies. Finally, it was agreed to use a technique to remove the liquid from the baby's brain prior to delivery and then to induce labour.

This baby's birth was a very traumatic experience; the baby's appearance was very abnormal, with her pyramid-shaped head, 2.5 litres of liquid having been removed from her brain, and a rib emerging from her belly. Both parents and the birth attendants were frightened, so much so that the mother regretfully recalled that ‘I did not even kiss her.’ The baby was named, lived for less than an hour and subsequently was buried in the Muslim cemetery.

During the pregnancy, the couple had considered abortion. As Shakeela explained during an interview:

I thought I might, even before the scan showed the fluid on the brain … My husband said he would support me, but my Mum said in our religion termination is a sin, it is murder and God will punish you after death, unless it is certain the mother will die … otherwise it is not allowed. My Mum said she would look after the baby if it is handicapped … My Dad told me to pray. He said, ‘the doctors aren't always right. These things are in the hands of God’.

Shakeela also consulted her sister. Her sister informed her that their sister-in-law, who is married to an *imām* (Muslim priest) who received his religious training from Al-Azhar University in Egypt, had terminated her third pregnancy after scans showed fatal abnormalities in the foetus. However, her sister-in-law said she did not wish to discuss the matter. Shakeela then consulted books on Islam in her parents' home. These confirmed the opinion that abortion is only permissible if the mother's death is certain, for then the baby would have no mother. She also enquired at the public library, and found some books describing British women's experiences of pregnancy loss, abortion and infant death, but nothing that addressed her dilemma as a Muslim. Finding no Islamic support for doing so, she did not end this pregnancy.

However, Shakeela went on to say that, at the baby's funeral, she heard from a woman offering condolences that there are alternative views on abortion in Islam that permit abortion within the first 120 days (4 months) of pregnancy and after 120 days if the foetus is expected to be seriously deformed:

That's when I realized why my sister-in-law had an abortion, but she didn't want to explain this to her parents or to my parents. When I told my Mum my sister-in-law had done it, my Mum said, ‘*Istighfar*, God have mercy’. It's a shame people don't discuss these things. Why don't they discuss it on digital or on TV? Or even have some leaflets about Islam and abortion. So many people don't know what to do. They say Islam has a solution for everything, so why are there no guidelines for this?

## DISCUSSION

Very few study participants acknowledged that there is a range of Islamic views on the acceptability of abortion, including the view that abortion is acceptable prior to 120 days (4 months) for ‘certain specific reasons’ and after this on grounds that include serious or fatal abnormality.^7^ The dominant local view, that abortion is only permissible in circumstances that threaten the mother's life, reflects the fact that obtaining information about the medical status of the embryo or foetus has only become a recent possibility, as a consequence of technological innovation. The range of views on the permissibility of abortion within Islamic scholarship also reflects the fact that the moral status of the foetus in Islamic law is contested, because the beginning of life is imprecisely defined in the Qur'an and Hadith.^8^ There are differing interpretations of Qur'anic verses concerning the developmental status of the foetus, at 40-day intervals^9^ and of an authenticated Hadith that suggests ‘ensoulment’ at 120 days significantly transforms the foetus into a human being – a ‘person’. Ensoulment occurs when angel breathes the spirit or soul into the foetus.^10^ Most interpretations permit abortion after 120 days (10 weeks) only if the foetus threatens the mother's life, if the pregnancy is harming an already suckling child and if the foetus is expected to be seriously deformed.^11^

The lack of consensus on abortion before 120 days reflects concerns to respect the potential for human life and the well-being of the foetus in relation to that of the mother, family and wider society. The legal positions comprise (i) unconditional permission; (ii) conditional permission for an acceptable medical or social reason; (iii) strong disapproval and (iv) unconditional prohibition. Foetal abnormality is sometimes included among acceptable reasons for terminating a pregnancy within the first 120 days. For instance Kuwait and Saudi Arabia have issued religious rulings (Fatwas) that include foetal abnormality among the reasonable grounds for abortion before 120 days.^12^ Iran has issued Fatwas permitting abortion before 10 weeks following a positive test for thalassaemia, and also within the first trimester of pregnancy (up to 13 or 14 weeks) for reasons that include the mother's health and foetal abnormality.^13^ In Pakistan there have been no equivalent state-authorized Fatwas, but authoritative statements by Muslim scholars can influence parents' decision-making in the light of a diagnosed abnormality, for example by reassuring couples about the permissibility of using prenatal testing and abortion for thalassaemia.^14^

Among the study participants, the dominant view was that in Islam abortion is unconditionally wrong, that ‘our religion does not allow’. This was sometimes expressed defensively as an illustration of the difference between Muslims and non-Muslims; as one woman said, ‘English people will do it if having a baby is not convenient. We do not do this’. However, the dominant stated view that abortion is unconditionally unacceptable does not mean that in practice Muslim women and girls never consider or never have abortions, or that medical staff should not raise the subject of abortion where it is indicated as a medical option. On the contrary, Pakistani Muslim women do sometimes have abortions, for social, psychological and medical reasons. One study participant had an abortion on the grounds that doctors predicted the baby would certainly die. Several study participants said that they would consider abortion if prenatal tests showed serious abnormality, and would do so for personal or medical rather than religious reasons. When asked if the timing of a prenatal test was important, one husband replied that it was not because their decision would be a medical decision, abortion being forbidden in Islam.

In consequence, the experience of abortion is frequently private and accompanied by guilt and strong feelings of wrong-doing. It is rarely discussed directly. Couples may simply tell their relatives that they lost the pregnancy, saying in Urdu or Punjabi that ‘the baby fell’. Some women decide to abort an abnormal foetus without the support of a husband or in-laws, occasionally under threats of bodily harm. The experience of abortion can render women vulnerable to malicious gossip within the neighbourhood. Doctors may arrange for abortions to take place in another hospital, distant from the local one, for this reason. Within the locality, stories circulate about women who have had abortions and then been punished by Allah by subsequently giving birth to children with psychiatric problems or physical disabilities, or being subsequently shown that their aborted foetus was perfectly healthy. Since the dominant view is that ‘the kind of child you have is up to Allah’, aborting an abnormal foetus may be interpreted as indicating a lack of strength to meet the challenges of God's will. These views can contribute to the psychological distress women can experience after having an abortion.

## CASE 2

This case concerns another UK-raised woman, Sameena, married to a cousin from Pakistan, whose first baby was born by Caesarean section at 36 weeks and lived for 3 days. This baby received *azān* spoken by Sameena's father soon after it was born. *Azān* (Arabic: *adhān*) refers to the words of the Muslim call to prayer which are customarily spoken into the newborn baby's ear by a senior male relative, or an *imam* (Muslim priest) as a marker of Muslim identity. The baby also received *janāza* (the Muslim funeral) and was buried in the Muslim section of the local cemetery. Her second baby, however, was born dead at 40 weeks and so did not receive *azān* and, because it had not been marked as a Muslim, was not entitled to *janāza*. It was buried in the snowdrop garden of the public cemetery, which is designated for miscarried or stillborn babies, rather than in the Muslim section of the cemetery. In her third pregnancy, there was no sign of abnormality, but Sameena wanted to have a Caesarean delivery, ‘to get the baby out early, while it is still breathing’, and to ensure that her father would be present to give *azān*, in case the baby stopped breathing. This was a source of considerable anxiety to her, which she eventually raised with her doctors. Her doctors explained that they were unwilling to perform Caesarean sections without good medical indicators, because of the risk of complications for the baby and the mother, and because of the added risks of complications for the mother in subsequent pregnancies. They also struggled to understand why Sameena was so anxious that her baby should draw breath: was this a cultural or a religious issue?

## DISCUSSION

In Sameena's view, a stillborn baby or a baby who has stopped breathing cannot receive *azān* and consequently cannot receive *janāza* (Muslim funeral) and be buried in the Muslim section of the local cemetery. Other study participants shared this view, which would seem to imply that, for them, receiving *azān* is central to defining Muslim personhood. Several other parents of babies unlikely to live long shared this concern; Shakeela, for example (Case 1) had also worried that her father would not reach the hospital in time to perform *azān*.

Regarding *azān*, Islamic scholarship holds that a newborn baby must give some sign of life by crying or breathing before receiving *azān*, but that if it is born alive and dies within seconds then *azān* is inappropriate. This conforms to the view expressed by study participants that the baby must be alive to receive *azān.* However, although Islamic jurists recommend the father recites *azān* into the baby's right ear, and *iqama* into its left, it is not obligatory for the father to do this, and *azān* can be spoken by anyone, including the baby's mother. This suggests that waiting for the arrival of a senior male relative or an *imam* is ‘customary’ rather than a religious requirement, a point that may be particularly relevant for cases in which doctors predict the baby will not survive.

*Azān* appears, therefore, to be a marker of the start of one's life after birth as a Muslim person; it would seem, therefore, to be inappropriate where there are clear medical indications that the baby will not survive. However, within Islamic scholarship, it does not necessarily follow that a stillborn or dead baby born to Muslim parents is not a Muslim, or that receiving *azān* is a necessary prerequisite for *janāza*. *Janāza* comprises washing the body and wrapping it in cloth, reciting *janāza* prayers and, in local practice, burial in the Muslim section of the local cemetery. According to Islamic scholarship, the washing of a dead baby's body is obligatory if the baby has shown some sign of life, and recommended even if it has not, provided the body is complete; if the body is incomplete, then it should be simply be wrapped and buried. *Janāza* prayers and burial in the Muslim cemetery are appropriate and recommended for a stillborn baby or a baby who was born alive but died before receiving *azān*. Moreover, one school of Islamic law (the Shafi'i school) holds that *janāza* prayers can be recited for a very premature (born before 6 months) or stillborn baby if it has shown some sign of life before birth. In all cases, it is also recommended that the baby is named, because the person will be called by their name at the day of resurrection.

As for burial, Islamic scholarship holds that burial can be done anywhere while socio-custom, tradition and local socio-economic factors, including cost of land, may exert significant constraints in practice. In the UK, Muslim burials take place within designated sections of local public cemeteries and in theory any Muslim who dies may be buried in the local Muslim cemetery, including (according to the Shafi'i school, at least) a stillborn baby or miscarried foetus born to Muslim parents. Thus, the burial of stillborn and miscarried babies outside the Muslim section of the local cemetery may reflect local socio-economic constraints combined with the traditional or customary belief that *azan* is a necessary pre-requisite for burial within the Muslim section of the cemetery.

Local women's concerns over whether or not their newborn baby will breathe in time for *azān* seem, in my view, to reflect deep anxieties about the baby's religious identity and, connected with this, the social invisibility of the parents' loss of the baby. One UK-raised woman whose husband is from Pakistan said of her stillborn son:

If he had drawn even one breath, then we would have had a funeral, all the family would have been there … but no, it's like he never existed. One Pakistani couple, I heard, their baby was born dead, they just left it in the hospital.

Another UK-raised woman whose husband is a cousin from Pakistan was very distressed by her family's response to her baby being born dead:

My husband and parents would not recognize him as a person to be named because he had not drawn breath. So I named him myself. He is buried in the snowdrop garden.

My interpretation of these concerns expressed by British-raised Pakistani women is that they reflect changing ideas about the social and psychological significance of infant death, the status of a newborn baby, and the management of stillbirth. Anthropological evidence suggests that in societies where infant mortality is high, miscarriage and stillbirth receive little social acknowledgement, and may be spoken of with a lack of emotion, despite the distress to women.^15^ Some of the study participants told me that, in rural Pakistan, miscarried or dead babies are left outside the village, while, apparently, in cities such as Lahore such babies are buried within Muslim cemeteries, in designated areas. This difference may reflect changes in practice regarding the recognition of stillbirth and miscarriage, and perhaps also differences in infant mortality rates within Pakistan. Similar changes have occurred in Europe over the past few generations. In Ireland, until the 1960s, un-baptized infants and children were not buried in churchyards because the Catholic Church did not regard them as part of the community and so there was no public funeral. For parents, this meant there were no visitors to the home offering condolences, no funeral, and thus no social recognition of their loss; women who recalled their experiences of pregnancy loss at that time said they had not even talked to their daughters about it.^16^ Today, in contrast, in Ireland and elsewhere in the UK, professional emotional support is offered to parents, and the social recognition of a baby's death is encouraged through practices such as naming the baby and creating photographic ‘memory books’. Additionally, changes in burial practice through the allocation of sections of local cemeteries for the burial of miscarried or stillborn babies mean that formal or public recognition is given for the of the loss of the baby, as a person. This formal process of acknowledgement of the parents' loss is both socially and emotionally important.^17^ While the general UK hospital practice now is to recognise stillbirths and miscarriages as losses of persons, through practices such as burial in a ‘snowdrop garden’, it seems from the cases discussed here that this recognition is not neccessarily shared by all Muslim parents. Within the snowdrop garden itself, the mounds of earth indicating the Muslim graces were identifiable because they faced Mecca, but very few of these graves had name plaques or flowers, and were in most cases overgrown and untended, in contrast to the non-Muslim graves.

## CASE 3

Talib is UK-raised father, married to a cousin from Mirpur, whose second baby had been kept alive in SCBU (the special care baby unit) for three weeks until the doctors asked Talib if the medication and ventilation should continue. Aware that of the Qur'anic injunction against killing one's children, Talib was uncertain of thereligious ethics of discontinuing life support. He consulted a *pir* (saint, or religious expert) known to his family for generations, who happened to be visiting from Mipur at the time, and asked this *pir* to come to the hospital with him to see the situation for himself before offering advice:

He stayed there for about half an hour. I think he was disturbed by what he saw. He said he thought it would be wrong to make her die, but it would be wrong to increase her suffering. So I said to the doctors, just keep the tubes and medicines, but … no ventilator now. It's up to her, we will see if she has the will to live … And … I could see … it was too much for her.

Having discussed the matter with a religious authority whose opinion he could trust, Talib felt able to live with his decision; talking to his *pir* had lessened his burden. However, he simply told his family, including his wife, that ‘the baby died’. Moreover, in a subsequent interview he asserted that withdrawing life support is unconditionally wrong in his religion, and he asked for reassurance that his identity would not be revealed.

## DISCUSSION

Three other couples in this study confronting similar situations made the same decision, and they also simply told the rest of their family that ‘the baby died’, lest they be accused of permitting the killing of their baby.

According to Muslim scholarship, the Qur'anic injunction against killing one's children^18^ can be interpreted in historical context as a response to the pre-Islamic practices of burying alive female infants and thus as condemnation of mysogyny and infanticide, and not of such practices as the use of birth control.^19^ In relation to the withdrawal of life support from babies in special care, various Islamic legal scholars consider it is acceptable to disconnect life support when the brain stem dies, as this is when the soul is thought to leave the body. Moreover, ‘in this case, the patient should be allowed to die, and thus any attempt to try to prolong life would be tantamount to punishing the patient’.^20^ In other words, Talib did the correct thing according to Islam and his *pir* gave the correct advice. Talib's subsequent private disquiet was striking; he seemed haunted by the idea that he might, nonetheless, have done the ‘wrong’ thing, or that, even if his personal conscience is clear, his decision would be strongly disapproved of by other members of his community if they knew about it.

## CONCLUSIONS AND IMPLICATIONS

Islamic law is a contested domain; there is ‘much debate … on almost every topic’ even among the scholars who produce *fatawa*s.^21^ This debate involves a process of interpretation and reasoning that seeks to relate the revealed sources to the circumstances of Muslim life, and as a result ‘ethics constantly changes as it relates to circumstances, which are seldom static’.^22^ The pre- or neo-natal diagnosis of fatal abnormalities confronts many parents of Pakistani origin with dilemmas that they are unlikely to have faced in rural Pakistan.^23^ In the cases discussed here, the dominant local understanding of Islamic ethics appears to be to prioritize ‘life’, although life has differing definitions, as ‘ensoulment’ at 120 days, and as ‘breathing’ at birth, permitting *azan* and *janaza*, or with the support of cardio-respiratory apparatus. Parents' responses in these situations reflected the dominant family and local community opinion about what is right as well as customary practice. In relation to abortion and withdrawal of life support, parents clearly felt the need for ‘backing’ in the form of an authoritative religious opinion where there was potential conflict with dominant local views. Some also experienced private unease or guilt that they had done wrong, or felt compelled to conceal their decision for fear of disapproval from relatives and other community members, even if their decision had been a responsible one that balanced different potential harms.

Having more information about the circumstances in which Islam permits abortion and withdrawal of life support from infants in special care, and having more open discussion of the emotional and social significance of public recognition of stillbirth or miscarriage would be helpful for parents and health-service providers supporting parents in making fully-informed decisions. Sometimes, medical personnel do not raise the topic of prenatal testing and abortion with Muslim patients, believing that there is no point in doing so because abortion is forbidden in Islam.^24^ This prejudging of patients' religious views regarding reproductive decisions undermines the principle of equality of access to health services. Patients should have the opportunity to consider all their reproductive options, and to make informed choices about their use. These choices may or may not take their faith into account. Some parents justify their decisions as ‘medical’ rather than based in religion, saying that ‘we will do as the doctors say’ when considering abortion or that a prenatal test result before 120 days is unnecessary because an abortion decision would be a ‘medical decision.’ Recent studies of Pakistani Muslim women's attitudes to prenatal diagnosis and termination of pregnancy indicate that ‘religion should not be taken as a proxy for their attitudes either for or against termination of pregnancy’.^25^ Parents who have had a child with a genetic problem and know they have a recurrence risk make decisions about prenatal diagnosis and abortion that reflect the circumstances of their lives, marriages and family, and may or may not include religious considerations.^26^ These studies challenge stereotypes about the role of cultural and religious difference in reproductive decision-making. Nonetheless, this paper suggests that sometimes describing a decision as ‘medical’ conceals concern that the decision is ‘wrong’, according to Islam. This paper has identified several areas in which more information on the Islamic positions regarding abortion and the withdrawal of life support would help parents and families in making fully informed decisions.

## Footnotes

1 A. Shaw. 2009. *Negotiating risk: British Pakistani experiences of genetics.* Oxford: Berghahn Books; A. Shaw &J.A. Hurst. ‘What is this genetics, anyway?’: Understandings of genetics, illness causality and inheritance among British Pakistani users of genetic services. *J Genet Couns*, 2008; 17; 4: 373–382; A. Shaw &J.A. Hurst. ‘I don't see any point in telling them’: attitudes to sharing genetic information in the family and carrier testing of relatives among British Pakistani adults referred to a genetics clinic. *Ethn Health*, 2009; 14; 2: 205–224.

2 Shaw & Hurst, 2008, *op. cit.* note 1.

3 A. Shaw. The arranged transnational cousin marriages of British Pakistanis: critique, dissent and cultural continuity. *Contemp South Asia*, 2006; 15; 2: 209–220.

4 K. Hasan. The medical and social costs of consanguineous marriages among British Mirpuris. *South Asia Res*2009; 29; 3: 275–295.

5 Shaw, *op. cit.* note 3; Shaw, *op. cit.* note 1; Shaw & Hurst, 2008, *op. cit.* note 1.

6 Shaw, *op. cit.* note 1; Shaw & Hurst, 2008, *op. cit.* note 1; Shaw & Hurst, 2009, *op. cit.* note 1.

7 D. Atighetchi. 2007. *Islamic Bioethics: Problems and Perspectives.* The Netherlands: Springer: 91–133; 250–254; L. Hessini. Abortion and Ethics: Policies and Practice in the Middle East and North Africa. *Reprod Health Matters*2007; 15; 2: 75–84; K.M. Hedayat. P. Shooshtarizadeh &M. Raza. Therapeutic abortion in Islam: contemporary views of Muslim Shiite scholars and effect of recent Iranian legislation. *J Med Ethics*, 2006; 32; 11: 652–657; V. Rispler-Chaim. The right not to be born: Abortion of the disadvantaged fetus in contemporary fatwas. *Muslim World*, 1999; 89; 2: 130–143; V. Rispler-Chaim. 1993. *Islamic Medical Ethics in the Twentieth Century.* Leiden: Brill: 8.

8 A. Sachedina. 2009. *Islamic Biomedical Ethics: Principles and Application*. Oxford: Oxford University Press: 125.

9 Qur'an 39:6; Qur'an 23; 12–13, quoted in S. Shaikh. 2003. Family planning, contraception, and abortion in Islam: undertaking *khilafah*. In *Sacred Rights: the case for contraception and abortion in world religions.* D.C. Maguire, ed. Oxford: Oxford University Press: 120; see also A.I. Al Aqeel. Islamic ethical framework for research into and prevention of genetic diseases. *Nat Genet*2007; 39:11: 1293–1296; V. Rispler-Chaim, 1993, *op cit.* note 7; A.F.M. Ebrahim. 1988. *Biomedical issues: Islamic perspective*. Mobeni: The Islamic Medical Association of South Africa: 114–115.

10 Muslim, Book of Qadr, Hadith no 4781, quoted in Shaikh, *op. cit.* note 9, p.120; see also Ebrahim, 1988, *op. cit.* note 9, pp. 115–116.

11 A.R. Omran. 1992. *Family Planning in the legacy of Islam*. London: Routledge: 192. See also Atighetchi, *op. cit*. note 7, pp. 91–133; 250–254; and D.L. Bowen. 2003. Contemporary Muslim Ethics of Abortion in J.E. Brockopp, ed. *Islamic Ethics of Life: Abortion, War, and Euthanasia.* Columbia: the University of South Carolina Press: 51–80.

12 M.A.S. Abel Haleem. 1993. Medical ethics in Islam. In *Choices and Decisions in Health Care*. A. Grubb, ed. Chichester, UK: John Wiley and Sons: 1–20; F.S. Alkuryay &R.A. Kilani. Attitudes of Saudi families affected with haemoglobinopathies towards prenatal screening and abortion and the influence of religious ruling (fatwa). *Prenat Diagn*2001; 21: 448–451. See also Atighetchi, *op. cit.* note 11, pp. 91–133; 250–254; L. Hessini, *op. cit.* note 7.

13 Shaikh, *op cit.* note 9, p. 122. See also Hedayat, Shooshtarizadeh & Raza, *op. cit.* note 7.

14 S.M. Ahmed *et al*. Prenatal diagnosis of beta-thalassaemia in Pakistan: experience in a Muslim country. *Prenat Diagn*2000; 20: 382.

15 R. Cecil. 1996. Memories of pregnancy loss: recollections of elderly women in Northern Ireland. In *The anthropology of pregnancy loss: comparative studies in miscarriage, stillbirth and neonatal death.* R. Cecil, ed. Oxford: Berg: 177–195; P. Jeffery & R. Jeffery. 1996. Delayed periods and falling babies: The ethnophysiology and politics of pregnancy loss in rural North India. In *The anthropology of pregnancy loss: comparative studies in miscarriage, stillbirth and neonatal death.* R. Cecil, ed. Oxford: Berg: 17–37.

16 Cecil, *op. cit.* note 15.

17 P. Connerton. 1989. *How societies remember*. Cambridge: Cambridge University Press.

18 Qur'an 6:151, quoted in Shaikh. *op. cit.* note 9, p. 113.

19 F. Rahman. 1982. The Status of Women in Islam: A Modernist Interpretation. In *Separate Worlds: Studies of Purdah in South Asia.* H. Papanek & G. Minault, eds. Delhi: South Asia Books: 286–287.

20 A.F.M. Ebrahim. 2001. *Organ Transplantation, Euthanasia, Cloning and Animal Experimentation: An Islamic View*. Leicester: The Islamic Foundation: 95.

21 Rispler-Chaim, 1993, *op. cit.* note 7, p. 4.

22 Ibid: 3.

23 Shaw, *op. cit.* note 1.

24 B. Modell et al. Informed choice in genetic screening for thalassaemia during pregnancy: audit from a national confidential enquiry. *Br Med J* 2000; 320: 337–341.

25 S. Ahmed *et al*. The influence of faith and religion and the role of religious and community leaders in prenatal decisions for sickle cell disorders and thalassaemia major. *Prenat Diagn*2006; 26: 801–809; S. Ahmed *et al*. Antenatal thalassaemia carrier testing: women's perceptions of ‘information’ and ‘consent’. *J Med Screen*2005; 12: 69–77; J.M. Hewison *et al*. Attitudes to prenatal testing and termination of pregnancy for fetal abnormality: a comparison of white and Pakistani women in the UK. *Prenat Diagn*, 2007; 27: 419–430.

26 Shaw, *op. cit.* note 1.

